# Conspecific recognition of pedal scent in domestic dogs

**DOI:** 10.1038/s41598-020-74784-5

**Published:** 2020-10-20

**Authors:** Kari McClanahan, Frank Rosell

**Affiliations:** Department of Natural Sciences and Environmental Health, Faculty of Technology, Natural Sciences and Maritime Sciences, University of South-Eastern Norway, Bø in Telemark, Norway

**Keywords:** Ecology, Zoology

## Abstract

Carnivores rely heavily on scent to communicate with conspecifics. Scent glands located on the underside of the feet provide an especially efficient way of leaving a scent trail. Although domestic dogs (*Canis familiaris*) are well-known for their olfactory abilities and scent marking behaviours, their use of pedal scent for communication remains unknown. We studied the reaction of intact dogs of both sexes to male and female pedal scent as well as a control sample of scent taken from the ground, using sniffing time and nostril usage as an indicator of interest level and emotional valence. In male subjects, only the sniffing duration for other males differed from the control samples, with no clear difference detected between male and female scent. Females showed no difference in the sniffing duration for any sample type. Conversely, male nostril use did not differ between the sample types, whereas females demonstrated a right nostril bias when sniffing the scent from other females and a left nostril bias when sniffing the control. We have shown that dogs recognize scent taken from the pedal glands from other dogs, although the extent to which they use this information to determine the sex of the scent depositor remains unclear.

## Introduction

Olfactory communication through pedal scent glands, which includes the foot pads and interdigital regions, provides an efficient means for mammals to convey information with a low energy cost. By depositing scent passively while they walk, the signalers minimize the cost of communication and leave a continuous trail, which enhances the likeliness of being encountered^1^. The eccrine glands in the footpads of domestic cats (*Felis catus*), raccoons (*Procyon lotor*) and wolves (*Canis lupus*), for example, are used for territorial scent marking activities, tracking abilities and orientation^2–4^. Interdigital apocrine glands have been described in several ungulate species as being used for communicating information about reproduction, territorial demarcation and social behaviour^5^.

One important use for the scent deposited through the feet is distinguishing between sexes. For example, reindeer (*Rangifer tarandus*) can use the scent left by interdigital glands to find their way back to their herd, and show a preference for same-sex scent during the early summer when the males and females are segregated^6^. Polar bears (*Ursus maritimus*) spend significantly more time investigating interdigital scent taken from the opposite sex, and likely use this information when searching for a mate^1^. A chemical analysis which found sexual dimorphism of the compounds from pedal secretions in brown bears (*U. arctos*) offers further support for this observation^7^. In the North American river otter (*Lontra canadensis*), the male possesses glands on the inner pads of the toes on the hind paws which are absent in the female, indicating a sexual function^8^.

Olfactory communication is particularly prevalent in carnivores^9^, and the wolf’s pack-living and territorial nature make communication via scent a useful way to obtain information about group members and to assess potential threats from conspecifics while avoiding direct conflict^10^. Ascertaining the sex of other wolves may be of particular value, as the Ethiopian wolf (*C. simensis*) is more tolerant of opposite sex neighbors than those of the same sex^11^, and male grey wolves are more likely than females to chase away rival packs or lone male wolves^12^. For lone wolves, the ability to read the sex from scent marks left by other wolves facilitates locating a mate and forming a pack^13^.

Although the domestication process has altered their behaviour in addition to their morphology, as descendants of the grey wolf, the domestic dog (*C. l. familiaris*) has retained many characteristics which may help to explain their social behaviour^14,15^. Like the wolf, the dog is well-known for its use of olfaction^16^, and several studies have demonstrated their ability to distinguish between sexes using urine^17–19^. Further, chemical analysis has demonstrated sexual dimorphism in the composition of anal gland secretions^20^. There may be several reasons for a dog to ascertain the sex of another dog. For males, other males are more likely to be an aggressor than females^21^, so male scent may warrant investigation to evaluate the potential threat. Female scent may be of interest to male dogs as they evaluate their potential for breeding^17^. For female dogs, other females are more likely to be aggressors^22,23^. With the high energy costs of producing offspring, female mammals often compete with each other for resources, which can lead to high levels of aggression^24^. However, males are generally more able to outcompete females for resources, irrespective of their sizes^25^. Therefore, females may regard either sex as a threat.

The presence of scent producing glands on the foot pad and interdigital region in dogs suggests an olfactory component which may be useful in communicating with conspecifics^4^. These scents are believed to be used in marking territory and producing alarm signals^26^. Dogs are frequently observed scratching the ground after urination or defecation, which may deposit interdigital scent as well as serve as a visual cue^27^. Although there is speculation that odours from the feet may be used for individual recognition^28^, no studies have been done to investigate its potential for sexual discrimination. However, the secretions of apocrine glands which are present between the toes derive their scent from the microflora communities present on the skin^29^, and have been shown to vary in composition between sexes, and therefore may convey this information^30^.

Understanding an animal’s emotional response to a scent can be facilitated by the proposal that emotional reactions are lateralized in the brain, with the right side involved with sympathetic activation, or aversive response, and the left hemisphere with parasympathetic activity associated with reduced tension or calm responses^31^. In mammals most receptor information from each nostril projects to the primary olfactory cortex in the same hemisphere^32^. Siniscalchi, et al.^33^ found that dogs primarily used their right nostril to sniff adrenaline and sweat from a veterinarian, whereas they used their left nostril to sniff nonaversive odours such as food, lemon, vaginal secretions, and a cotton swab. In addition, they initially use their right nostril when sniffing a novel stimulus, but switch to their left nostril for nonaversive stimuli; when sniffing aversive stimuli they continue to favour their right nostril^33^.

We hypothesized that pedal gland secretions code for sex in the domestic dog as in other carnivores. We predicted that males would spend more time sniffing the scent from other males and to favour their right nostril as they evaluate the potential threat. On the other hand, we predicted males to favour their left nostril when sniffing the scent from females, as this is expected to be a more pleasant stimulus. We predicted that female subjects would equally spend more time sniffing male and female scent compared to a control, and to favour their right nostril for scent from both sexes as either sex may pose a threat.

## Results

### Sniffing duration

**Figure 1:**
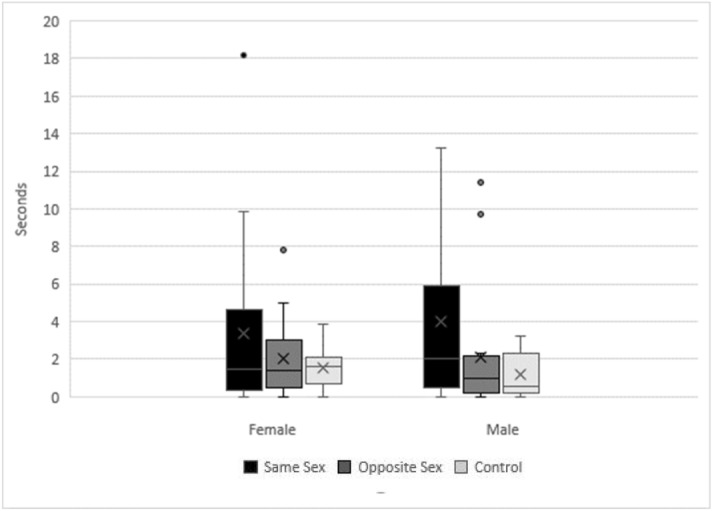
The amount of time in seconds that dogs spent sniffing pedal scent samples from their own sex (black), the opposite sex (medium gray), and control (light gray). Error bars represent the standard error of the mean (SEM).

Dogs sniffed all samples for an average of 2.51 s (median = 1.45; SD = 3.30seconds). There was no difference in the overall sniffing duration between female and male subjects (β = 0.214, 95% CI: − 1.083, 1.511). The median sniffing time for male subjects was 2.55 (SD = 4.59) seconds for male scent, 0.98 (SD = 3.47) seconds for female scent, and 0.55 (SD = 1.15) seconds for the control sample. Males sniffed scent from other males longer than the control, although there was no clear difference in the sniffing duration between male and female scent (Table 1, Fig. 1). The median sniffing time for female subjects was 1.55 (SD = 1.98) seconds for male scent, 1.58 (SD = 4.81) seconds for female scent, and 1.73 (SD = 0.91) seconds for the control scent. Although females also tended to sniff the scent from their own sex the longest, no parameters were identified as informative for female sniffing duration (Table 1, Fig. 1).

**Table 1:** Effect size (β), standard error (SE), lower (LCI) and upper (UCI) 95% confidence interval of explanatory variables for male and female domestic dog sniffing duration and nostril use when investigating conspecific pedal scent.

Variable	β	SE	LCI	UCI
Male sniffing time				
Scent Sex (female)	0.229	0.472	– 0.725	1.183
** Scent Sex (male)**	**1.021**	**0.474**	**0.064**	**1.978**
Scent sex (male vs. female)^a^	0.812	0.474	– 0.300	1.924
Trial number	0.314	0.243	– 0.177	0.805
Female sniffing time				
Scent donor size (large)	0.675	0.379	– 0.085	1.434
Scent donor size (medium)	– 0.286	0.385	– 1.058	0.485
Scent donor size (small)	– 0.489	0.587	– 1.667	0.689
Trial number	– 0.222	0.193	– 0.609	0.166

A negative nostril indicates a right nostril bias associated with novel and/or arousal stimuli whereas a positive index indicates a left nostril bias associated with familiar neutral or pleasant odours. We performed model averaging of best models (ΔAICc < 2) to estimate the effect. Informative parameters are given in bold.

^a^We used a Tukey’s contrast to compare the sniffing duration for male and female scent.

**Figure 2:**
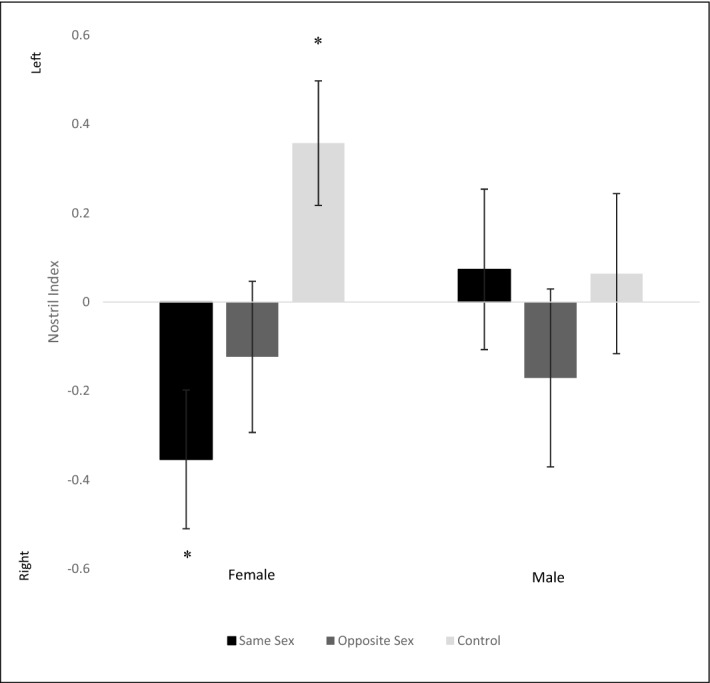
Nostril index for male and female subjects showing nostril use for same sex (black), opposite sex (medium gray), and control (light gray) with error bars representing the standard error of the mean (SEM). A negative index indicates predominant use of the right nostril, which is associated with arousal, while a positive index indicates use of the left nostril, or a more calming or neutral stimulus. Asterisks indicate a significant deviation from zero (*P* < 0.05, two-tailed one-sample Wilcoxon signed rank test).

**Figure 3:**
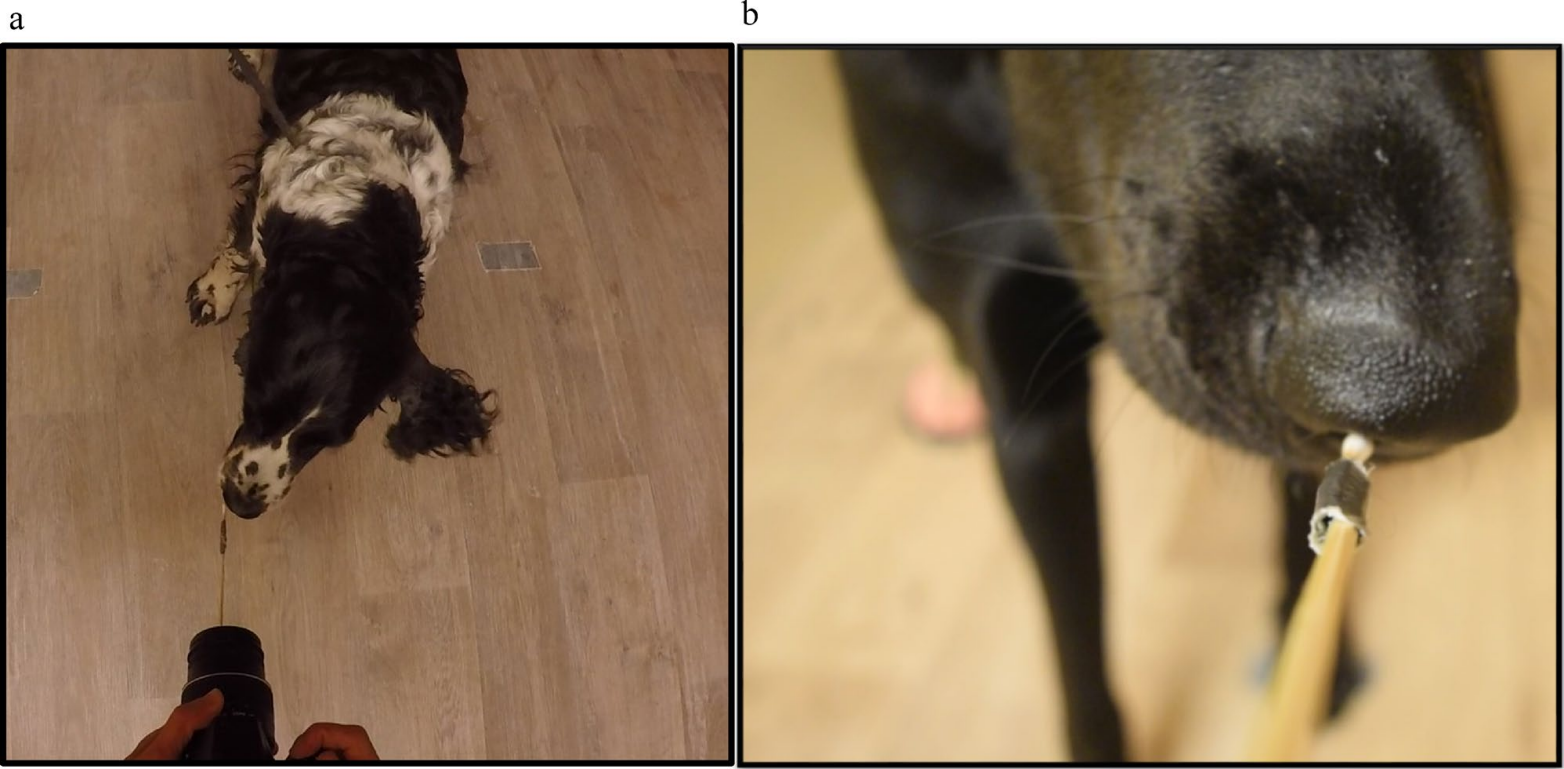
(**a**,**b**) The set-up of the sniffing aparatus used to test domestic dogs’ interest in the scent collected from the bottom of the feet for male and female dogs (**a**) and a view from the camera recording the dogs’ sniffing duration and nostril use for (**b**).

### Nostril index

For male subjects, no scent type resulted in a nostril index which significantly deviated from zero (two-tailed one-sample Wilcoxon signed rank tests, *P* > 0.05) (Fig. 2). The first and last nostril males used also did not deviate from chance for any of the sample types (binomial tests, *P* > 0.05). For female subjects, the nostril index for scent from other females had a significant right nostril bias (two-tailed one sample Wilcoxon test: Z = − 2.214, *P* = 0.027), and the control scent had a significant left nostril bias (Z = − 2.192, *P* = 0.028) (Fig. 2). There was no clear nostril bias for scent for male scent (Z = − 0.547, *P* = 0.584). Females started with their right nostril significantly more often than they started with their left when sniffing the scent from other females (Z = − 1.75, *P* = 0.025). The starting nostril for the other two scent types did not deviate from chance (*P* > 0.05). Similarly, they ended with their right nostril significantly more than their left nostril when sniffing scent from other females (Z = − 1.75, *P* = 0.038) with no difference in the other sample types (*P* > 0.05).

## Discussion

There was no clear evidence to support our prediction that male dogs sniff scent from other males longer than scent from females. However, only the scent from other males resulted in a longer sniffing duration than the control sample, indicating that this may have elicited a stronger response. Contrary to our predictions, male subjects did not show a nostril bias for any of the scent types. No clear difference was observed for female sniffing duration, however, they preferentially used their right nostril when sniffing scent from other females, and their left nostril when sniffing the control samples. However, no bias was observed for scent from males.

### Sniffing duration

Similar to our findings, Lisberg and Snowdon^34^ also did not find sex to have an effect on investigation time for urine from unfamiliar intact dogs. This is in contrast to Dunbar^17^ and Ranson and Beach^19^ who found that males investigate female urine more than urine from other males. However, Lisberg and Snowdon^34^ point out that unlike these previous studies, they used dogs which were not housed together, similar to our study design. Dogs living in the same group are already familiar with each other, so there may be less of a need to investigate members of their own sex olfactorily to assess potential competition or aggression, or to aid in establishing new relationships^34^ . In another mammal, the tuco-tuco (*Ctenomys talarum*), males were shown to spend similar amounts of time investigating both sexes, likely evaluating males as potential aggressors and females as potential mates^35^. These dual motivations for investigating conspecific scent may explain the similar sniffing duration observed in this study.

Another potential reason we did not find a clear difference in investigation time between the sexes is the source of the scent. In a study of investigation time in dogs, Dunbar^17^ found a preference for both males and non-oestrous females to investigate female urine longer than male urine, yet found no sex differences in the investigation of anal gland secretions, saliva, or ear wax. It may be that domestic dogs do not principally use these odours for determination of sex. With urine being a widely used method for communication, with deposits as frequent as twelve times per hour^36^ dogs may not have as much need to pay as close attention to odours from these other sources. Similarly, the golden hamster (*Mesocricetus auratus*) has been shown to discriminate individual scent from several sources (e.g. flank and ear gland secretions, vaginal secretions, urine and feces), but not from secretions in the feet^37^. There may be disadvantages to the signaler in advertising too much information. Rothman and Mech^13^ found that lone wolves scent mark at a lower rate than those in a pack, apparently in an attempt to avoid detection. It is possible that pedal scent glands do not convey information on sex to avoid automatically providing this information to potential rivals.

Although no difference between the sexes was detected, it is noteworthy that for male subjects only scent from other males differed from the control samples. In another group-living carnivore species, the African lion, males were found to respond more strongly to urine from other males than to urine from females, likely due to the intense competition males undergo for mates, as well as the need to keep track of companions^38^. Similarly, Rostain, et al.^39^ found that male river otters investigate the feces of other males significantly longer than feces from non-estrous females, and concluded that fecal scent plays a social role, as males are more social than females^40^. Conversely, it may be beneficial for an individual to investigate scent from a member of their own sex to determine the presence of a potential rival. In the brown hyena (*Hyaena brunnea*) members of the same sex were found to be agonistic towards each other, whereas members of the opposite sex often ignore each other^41^. Boydston, et al.^42^ suggest that a similar trend observed in the spotted hyena (*Crocuta crocuta*) may be the result of males defending their mates and females defending food and their offspring.

### Nostril index

Females more often used their right nostril when sniffing the scent from other females, which indicates that this elicits an arousal response. This can be related to emotions such as aggression, threat perception, or stress. In reviews of dogs being treated for aggressive behaviour, more females than males were found to start fights, and fights between two females led to more injuries than fights between two males or opposite sex pairs^21,43^. Thus, a female may become aggressive when encountering another female, or perceive her presence as a potential threat and seek to avoid her. In addition, females were found to suffer from more general anxiety and phobias than males^43^, to be less bold than males^44^, and to display more signs of acute stress^45^. Males on the other hand did not demonstrate a nostril bias for any of the sample types. In a study of free-ranging domestic dogs, male agonistic behaviours were most often observed in mating contexts and at territory boundaries, whereas females were more often agonistic in feeding contexts and in ‘other places’ (those outside of a feeding, mating, den, or territorial context)^46^. Because this study was done outside of a breeding context, males may not have exhibited as much negative associations with other dogs compared with females. In their wolf ancestors, male aggression towards other males primarily occurs during the breeding season, and interventions between other wolves are centered around those involving the alpha male’s partner, whereas female dominance displays occur throughout the year and show a more general intolerance to other females^47^.

Although domestic dog behaviour is largely shaped by their evolutionary history, the domestication process and their relationship with humans has introduced additional factors which influence their social behaviour. The individual dog’s temperament is highly influenced by its relationship with its human caretakers, who control most of a dog’s environment^48^. Factors such as the age at which the dog was brought into the household, the purpose for keeping the dog (i.e. working dogs vs. companions), the amount of time the owner spends with the dog, the number of people in the household, and even the education level of the owner can all influence the dog’s temperament and sociability towards other dogs^49^. This along with a dog’s socialization history likely affects whether a dog will consider the scent from another dog as a positive or negative stimulus, and likely contributes to the high variation in nostril index observed between dogs. This variation may also explain the neutral nostril index observed for both sexes in male subjects.

Siniscalchi, et al.^33^ have shown that dogs initially used the right nostril then switched towards the use of the left nostril when smelling non aversive stimuli, yet continued to use the right nostril when sniffing scents that caused more arousal. Females similarly ended with their right nostril more often when sniffing samples that contained female scent, indicating that this produced an arousal response. No such pattern was detected for males. One notable difference between their study and this one is the number of trials used for each scent. While we only tested each scent type once per dog, their study involved repeating the same scent for seven trials. It was only after between the fourth and sixth trial for each scent that the left nostril bias became evident. It is possible that had we repeated the same scent multiple times, a clear pattern would have also emerged for male subjects as they became more familiar with the scent.

Odours are made of a complex array of chemicals which we may not have been able to adequately account for. It may be that there are sex signals in the pedal glands but the dogs are responding more strongly to other cues such as the emotional state at the time of sample collection. Dogs may have been in varying emotional states prior to sample collection and had mixed reactions to having their feet rubbed, the stress of which may have been conveyed in the scent^50^. In addition, there may be differences between the subjects that we did not take into account. For example, Lisberg and Snowdon^34^ showed that dogs of a lower social status investigated conspecific scent for a longer duration than dominant dogs. Alternately, status may play a role in the scent, as only dominant wolves have been observed to engage in ground scratching behaviour^51^. In house mice (*Mus musculu*s) receptive females showed a preference for the scent from dominant males over the scent of subordinate males^52^.

## Conclusion

We have demonstrated that dogs respond more strongly to the pedal scents from other dogs of their own sex compared to a control, with males exhibiting a longer sniffing duration and females exhibiting a right nostril bias. However, it remains unclear as to what information is conveyed from pedal scents and how each sex uses this information. Future studies would benefit from including pedal scent from estrous females, as previous studies have had mixed results with male dogs’ interest in scent from non-estrous females.

## Materials and methods

### Subjects

Forty-eight domestic dogs (22 M, 26 F) living as household pets participated as scent investigators. Subjects were required to be at least 1 year old and averaged 6.0 years (SD = 3.3 years). None of the dogs had been neutered, as the 2010 Norwegian Animal Welfare Act considers this a prohibited procedure unless medically necessary. Dogs consisted of a mix of breeds of varying body sizes ranging from 3 to 35 kg and averaging 23.1 kg (SD = 7.7 kg). Dogs were recruited from a pool of participants from a previous study^53^ as well as from announcements on social media. All dogs resided within Vestfold and Telemark County, Norway with 40 in Bø and eight in neighbouring towns. Subjects also participated as scent donors for other dogs not living in their household.

### Scent collection

Ninety-six dogs served as scent donors (48 M, 48 F). They were also required to be at least one year old and averaged 5.0 years (SD = 3.4 years). They were a variety of breeds and sizes, ranging from 2 – 50 kg, with an average of 20.8 kg (SD = 9.4 kg). All females were non-oestrous as determined from interviews with the owners and none of the dogs had been neutered. Samples were collected throughout Vestfold and Telemark County as well as in Oslo County, approximately 110 km Northeast of Bø (n = 34 from outside Bø, 12 of which were from Oslo). Dogs were recruited from the same pool as the testing subjects, as well as from dog parks, dog clubs, and when encountered on their walks.

Scent samples were collected from the 28^th^ of February to the 11^th^ of June 2018 at the location dogs were encountered (e.g. parks and dog clubs) or at the lab when owners brought them in to investigate other samples. Scent was collected by rubbing a sterile cotton swab with a wooden core firmly against the metacarpal, interdigital, and area between the metacarpal pad and digits from the left hind paw. Because apocrine and eccrine glands are found on different parts of the foot^54^, these areas were chosen to capture the full range of scent that might be deposited as the dog walks, runs, or scratches the ground. One swab was used for each foot and rubbed firmly approximately five times over each area, so that each swab contained scent from all areas of the foot that were sampled. To reduce contamination from human scent, single-use vinyl gloves were used during collection and changed between each dog. Swabs were immediately broken above the cotton end, placed in a glass vial with a silicone lid (Fisherbrand Headspace-Vial, Precision Thread**)**, and stored in a freezer at – 20 °C. In instances when samples could not be placed in the freezer within an hour (n = 34) they were stored in a cooler which was packed with snow until they could be frozen.

### Experimental setup and procedure

The trials took place between 11^th^ May and 22^nd^ June 2018 in an empty 3.4 × 5.6 m room at the University of South-Eastern Norway Bø campus. Each dog investigated a male, female, and control swab which had been rubbed against the ground to collect environmental scents which are likely picked up by the dogs’ feet as they walk. All samples were used only once. The samples were removed from the freezer 30 min prior to the start of the session to come to room temperature, approximately 20 °C. Because the cotton swabs had been broken, they were taped to a wooden skewer which was taped to the bottom center of a digital camera (Nikon D3300; Nikon Corporation, Tokyo, Japan) to record the dogs’ nostril use (Fig. 3). The experimenter held the testing apparatus while kneeling at the center of the wall nearest the entrance, facing the dog^50^.

Each dog investigated the three scent types in a random order. Samples were prepared by an assistant out of view to create a double blind set-up in which both experimenter and dog owner were unaware of the identity^55^. Prior to the start of each session the dog was provided an opportunity to become familiar with the room and the experimenter (approximately 5–10 min), and trials began when the dog showed little interest in investigating the study room. The owner accompanied the dog into the study room as separation may cause the dog stress, and the owner’s presence creates a more natural situation for the dog^56^.

The trial began with the owner standing with the dog held loosely on a leash at a starting point marked on the floor, then proceeding along a designated path to a second mark where the experimenter was positioned with the video camera. Half of the owners stood to the left side of the dog while the other half stood to the right side to prevent the position affecting the dog’s performance^57^. Owners were also instructed to maintain a relaxed position and to avoid interacting with the dogs so as not to influence their behaviour. The experimenter held the video camera at the dog’s head height with both hands so as not to bias one side, and kept her eyes focused on the camera to avoid directing the dog’s attention in any one direction^58^, and also did not interact with the dog during the trial. Each trial lasted for 1 min with a 1 min interval between scents while the experimenter retrieved the next sample from the assistant in another room. The dog and owner returned to the starting position and repeated the procedure for each of the three samples. The experimenter washed her hands and the floor of the testing room with baking soda between each of the dogs^50^. Each dog participated in only one session consisting of the three scent stimuli.

### Ethical note

All methods were performed in accordance with the relevant guidelines and regulations of the University of South-Eastern Norway and no further permits for pet animals were required. Approvals from other ethics committees or ethics boards were not needed. No animals experienced anaesthesia, euthanasia or any kind of sacrifice as a part of this study. All dogs that contributed to this study had permission obtained from the owner.

### Data analysis

Only dogs which sniffed all three samples were included in the data analysis, and dogs which became distracted during the trials by sounds occurring outside of the room were excluded, leaving 34 dogs (19F, 15 M). An independent referee ensured that the experiments were double-blind^59^, and coded each video to the identity of the scent sample using frame-by-frame playback (60 frames per second) which were converted back into seconds to record the length of time the dog spent sniffing each sample. Sniffing was defined as the nose coming to within approximately 2.5 cm of the cotton swab^60^ accompanied by sharp inhalations through the nose. When subjects investigated the same sample multiple times, all durations were summed together. The time spent sniffing was recorded for each nostril, and when the cotton swab was directly between the nostrils the sniffing time was recorded but not attributed to either nostril.

Lateral asymmetries in nostril use was calculated using the index: LI = (L − R/L + R), where L and R indicate the total time in seconds spent sniffing with the left and the right nostril, respectively. A score of 1.0 indicates exclusive left nostril use and a score of – 1.0 indicates exclusive right nostril use. A score of 0 indicates equal use of the two nostrils^50^. After confirming that data was not normally distributed with a Shapiro–Wilk test, we used two-tailed one-sample Wilcoxon signed-rank tests to estimate significant departures from a neutral level of zero^33,50^. In addition, we used binomial tests to determine if the first and last nostril used for each sample type differed from chance frequencies.

To investigate the effects of sex on sniffing duration a linear mixed model (LMM) was used with sniffing duration as the dependent variable and the weight, age, and breed of the subject dogs and scent donors, the sex of the scent donor, the trial number, and whether the scent had come from a dog in Bø (used as a proxy for the potential for the subject to have previously encountered the dog or its scent) as fixed effects. The subject dog was included as a random intercept to take into account individual variations of sniffing patterns. Sniffing duration was ln-transformed to meet model assumptions of normally distributed residuals and homogeneity of variance. Due to the different maturation rates for dogs of different sizes^61^, age and weight were classified into categorical variables based on veterinarian recommendations^62^. Age categories included adult, senior, and geriatric, and size classes were divided into small, medium, large, and extra large. Breeds for the subject dogs and scent donors were determined by interviews with the owners and classified into seven groups based on the American Kennel Club group types^63^. Mixed breeds were considered to be the breed identified as being predominant. When covariates were found to be correlated (r > 0.6) separate candidate models were created and the one with the lowest Akaike’s Information Criterion corrected for small sample size (AICc) score was selected for the global model^64^. Correlation was observed between the size of the scent donor, the breed of the scent donor, the sex of the scent donor, and whether the scent donor lived in Bø. The dredge function from the package *MuMin*^65^ was applied to the global model to select the most parsimonious model based on the lowest AICc score^66^ (Table 2). Models with ∆AICc < 2 were averaged^67^ and parameters were considered uninformative if 0 was included in the 95% confidence interval^68^. A post hoc Tukey contrast was run from the package ‘multcomp’^69^ to make a pairwise comparison of informative parameters. All statistical analyses were performed in RStudio 1.1.463 (R Development Core Team 2015).

**Table 2:** The most parsimonious models (based on AIC weights) and the full model for the sniffing duration for male and female dogs smelling conspecific pedal scent from a male and female scent donor as well as a control over three random trials.

Model	Variables	df	logLink	AICc	Delta AICc	AICc weight
**Male sniffing time**						
1	Scent sex	5	– 75.636	162.8	0.00	0.230
2	Intercept only	3	– 78.146	162.9	0.07	0.222
3	Trial number	4	– 77.215	163.4	0.62	0.169
4	Scent sex + trial number	6	– 74.934	164.1	1.27	0.122
5	Subject age	5	– 77.563	166.7	3.86	0.033
Global	Subject age + scent donor age + scent dog sex + subject size + trial number + subject breed group	17	– 111.811	280.3	34.40	0.00
**Female sniffing time**						
1	Scent size	6	– 79.377	172.5	0.00	0.302
2	Intercept only	3	– 83.210	172.9	0.36	0.253
3	Scent size + trial number	7	– 78.834	174.1	1.56	0.138
4	Trial number	4	– 83.060	174.9	2.39	0.091
5	Subject age + scent dog size	8	– 78.767	176.7	4.19	0.037
Global	Subject dog age + subject size + scent dog size + trial number + subject breed group	21	– 68.503	207.9	35.34	0.000

## Data Availability

Appropriate data will be uploaded on Dyrad respository upon acceptance.
